# Skiagraphy in the Diagnosis of Chest Disease

**Published:** 1901-02-02

**Authors:** 


### SKIAGRAPHY IN THE DIAGNOSIS OF CHEST DISEASE.

At the last meeting of the Medical Society Dr. Hugh Walsham and Dr. Clifford Beale discussed the question of the value of skiagraphy in the diagnosis of diseases of the chest. It was shown that, either with the plate or the screen, it was absolutely necessary to employ a powerful coil capable of yielding a 12- or 14-inch spark. With such a coil an exposure of 2 minutes was as a rule quite sufficient, the tube being from 2 to 3 feet from the plate, and care being taken that the anti-cathode in the tube should be placed exactly vertically over the part of the chest to be examined. Of course, both with plate and screen—especially with the latter—proper allowance must be made for the excessive magnification of the parts nearest to the tube. No danger of dermatitis need be feared where the rays were only passed for a few minutes at a time. Cases had been reported from time to time, but in these cases the exposure had been excessive—viz., from half an hour to two hours. As to the diagnostic uses of the rays it was shown that in some cases tuberculous shadows could be detected before the presence of tubercle was indicated by any abnormal physical signs. The more advanced the disease the darker was the shadow, especially where caseation was in progress. Pus and caseation appeared to be the two conditions which were most clearly indicated by skiagraphy. There was a marked difference in the shadows produced in cases of simple and of purulent effusion in the pleura. But by far the most important uses of skiagraphy in chest disorders was in the detection of morbid growths and aneurysms, and many examples of the latter were shown in which the evidence given by the rays was far more precise than that obtained by ordinary methods. It was even possible to determine with considerable accuracy whether an aneurysm was full of dark clot or of semi-translucent fluid blood.

